# The People’s Doctors

**Published:** 1829

**Authors:** 


### REVIEWS AND BIBLIOGRAPHICAL NOTICES.

Art. XI.—The People's Doctors.—*New Guide to Health; or Botanic Family Physician.* Containing a complete system of practice upon a plan entirely new; with a description of the vegetables made use of, and directions for preparing and administering them to cure disease. To which is added a description of several cases of disease attended by the Author, with the mode of treatment and cure. By Samuel Thomson.—Horton Howard, Columbus, 1828. pp. 115.

*A course of fifteen Lectures on Medical Botany,* denominated Thomson's new theory of medical practice; in which the various theories that have preceded it, are reviewed and compared; delivered in Cincinnati, Ohio. By Samuel Robinson.—Horton Howard, Columbus, 1829.—pp. 200.

*Cases of cures performed by the use of Swaim's Panacea.* By Wm. Swaim, Philadelphia, 1827.—pp. 84.

*The Pulmist; or Introduction to the Art of curing and preventing the Consumption or chronic Phthisis.* A medical essay, including a new and better distinction of its causes, kinds, remedies, diets, and other peculiarities. By Professor Rafinesque, Ph. D. and Pulmist, Professor of Practical and Medical Botany, Natural and Civil History, &c. &c.—Philadelphia, 1829.

*The Compleat English Physician, or, the Druggists Shop Opened.*— Explicating all the particulars of which Medicines at this day, are Composed and made. Shewing their various Names and Natures, their several Preparations, Virtues, Uses and Doses as they are applicable to the whole Art of Physic, and containing above 600 Chymical processes.

A work of exceeding use to all sorts of Men, of what Quality or Profession soever. The *like* not hitherto extant.

In X. Books. Multa multumque. By William Salmon, Professor of Physic. London. pp. 1207.—1693.

---

"They shall have mysteries—aye precious stuff
"For knaves to thrive by—mysteries enough;
"Dark tangled doctrines, dark as fraud can weave,
"Which *simple* votaries shall on *trust* receive,
"While *craftier feign belief,* till they believe."

Robertson, the celebrated historian, expresses the opinion, that superstition 'was originally engrafted on medicine, not on religion.' However this may be, no one conversant with 'poor human nature,' can be unapprized of the close companionship which has always subsisted between them. In savage life, a belief in the healing efficacy of charms and incantations, is so universal, as to leave no doubt, that a principle of superstition is inherent in the human mind. It belongs, therefore, to man in every situation; though in civilized life its manifestations are comparatively few and feeble, for it is the tendency of all good education to limit its operations. Hence the most enlightened minds, display least propensity for the marvellous; but how can the intellect of a whole nation be cultivated on all subjects? This was never yet done, and we are sorry to add, never can be done. In despite of every exertion to illuminate the mass, many dark and impenetrable spots will remain; so that society, in its best composition, must continue to display enough of credulity to render it ridiculous. From the depths of ignorance, with its overshadowing superstitions,—when the hopes of the sick rest upon spells and coscinomancy,—the first step taken, is to blend with these *supernatural,* a variety of *natural* means, resting the efficacy of the latter, on the occult influence of the former. The next advance, leaves the mummeries of the sorcerer behind; but clings to amulets, seventh-sons, 'yarb-doctors,' and vagabonds. This brings us to our own age—than which, with all our boasted elevation in learning and philosophy, no other has ever presented a greater *variety* of barefaced and abominable quackeries. To eradicate them would be more difficult, than to root out the sour dock and Canada thistle of our fields, while the soil continues to favour its reproduction. Planted in the ignorance of the multitude, warmed by its credulity, and cherished by their artful and unblushing authors, these impostures are fixed upon us, as the 'poison oak' encircles the trunk of the noble tree, whose name it has prostituted. True it is, they are not always the same. The stupidest intellect at last comes to perceive their absurdity, and throws them off; but the impostors—

'New edge their dulness and new bronze their face,'

and speedily invent fresh draughts for the gaping and thirsty populace.

When one of these quackeries is inoculated into a community, nothing can arrest its spread, or limit its duration. Every dog has its day, and so has every nostrum. The gulping is universal; not extending, it is true, to every individual, but to all classes. The propensity to be cheated is not confined to men or women, the old or young, the poor or rich, the unlearned, or (we are sorry to add,) the learned; but displays its workings in the weak-minded and credulous of all. Like the small pox it prevails till all the susceptible are infected, and have gone through the disease. A moment of common sense may, perhaps, succeed to the period of suffering; as natural fools have sometimes spoken well from the shock of a violent blow. The desire to be cheated, however, returns apace; but not earlier than the desire to cheat—

"Then thick as locusts dark'ning all the ground,
"A tribe, with herbs and roots fantastic crown'd,
"All with some wond'rous gift approach the people,
"—Lobelia, pulmel, and steam kettle."

A new excitement now springs up. A blue light, such as the superstitious see rising from the church yard, spreads over the people, and reveals the *ecstacy* of every vacant and credulous countenance. Now is the time for dyspeptics and asthmaticks and hypochondriacks. Give them but a single draught. O how delightful! Perchance a ladle full from the chaldron of Macbeth; but no matter. Administered by wizzard hands it can do no harm. Down with the profession, *vive la Charlatanerie.* The world has been long enough duped by lawyers, and priests, and doctors. Let us rid ourselves of the last of them, if no more. If not the greatest impostors, they cheat us out of most money, and kill us to boot.—They bleed us to fainting, blister us to wincing, stupefy us with opium, vomit us with tartar instead of lobelia, salivate us with mercury, in place of the panacea, or the 'stone mason's balsam,' and, purge off with calomel all kinds of phlegm, but that which encumbers our brain! Let no one be over nice. The end sometimes justifies the means. Suffering humanity cries aloud, and must be rescued from the keeping of science and skill and professional charity. The world has been in error four thousand years; and the path of medicine may be followed back by the carcasses of its victims. *Doctor* Thomson, and *doctor* Swaim, and *doctor* Rafinesque have received new 'gifts,' and are ready to distribute them. Push aside the 'riglar' Doctors!—Conceal all their cures, and publish all their failures! Go among their patients, and labour to overthrow a long established confidence! Brand them with ignorance of the human system! Stigmatize them with cruelty! Denounce them as mercenary! and Libel them as infamous! Break down the *aristocrasy* of learning and science: Give the people their rights: let the drunken and lazy among the tailors, and carpenters, and *lawyers*, and coblers, and *clergy*, and saddlers, and ostlers, now rise to the summit level, and go forth as ministering angels! Become their patrons, and snuff up in turn the *steams* of their incense; sustain them against the professed Doctors; lecture them into notoriety: mould them into form as the bear licks her shapeless pups into beauty: turn jackals and procurers lest they might want business: stand responsible for their success: newspaper abroad their pretended cures; and handbill away the proofs of their murders! This being done—

> "The dawn will break upon us, and bright day shall go forth and shine; when we may hope to live with the dear objects of our love, until ripe and full of years, we shall be gathered to our fathers."
>
> *Robinson's Lect.* ix.

But we must withdraw from the view of these ravishing prospects, to examine some of the objects which lie in the foreground of the picture.

We need not tell our readers, that the mountebanks who are to bring about the aforesaid millenium; generally work on *patent* methods. This is indispensable to success—both in curing the sick, and getting the sick willing to be cured. It is edifying to see, how much of dignity and mystery are impressed on the vilest or the vapidest compound, by the great seal of state! Oh! to take medicine 'by authority' and that, too, of the President, who would recommend nothing that he had not tried on himself, and found useful for the people. He is the people's friend. He is a good doctor (not from 'book-larnin') but common sense; he once made a *great speech* in Congress, and that shows that he is a great *doctor*! before that he was a great *lawyer*, and of course is a great *doctor!* he fought a great *battle*, and is therefore a great *doctor!* It is a crafty error of the 'riglar' doctors, that a man should study their books to know how to cure diseases; for this knowledge, like 'reading and writing, comes by natur.' Let us stick then to the patents—they are 'genuine,' and contain no 'marcury.' Such is the fanaticism inspired in the multitude by the diplomas issued by the office at Washington! To trace out all its consequences, would carry us to the charnel house, which at the present moment, we do not feel inclined to enter. But what will the fanatics think, when we tell them, that neither the president, nor the secretary, nor even good old Dr. Thornton himself, ever ventured to swallow one of these precious boluses or pills, the 'panacea,' 'catholicon,' or 'pulmel;' 'skunk cabbage,' 'number 6,' 'clover heads,' or 'cayenne'—except when they have added the last to their roast beef, or turtle soup? We fear they can scarcely believe us, credulous as they are; but still such is the mortifying fact. The whole process for the invention, patenting, selling, and killing off by means of these preparations, may be stated in a few words. Pick up a physician's recipe for 'antibilious pills,' 'stomachic bitters,' or 'diaphoretic powders'—or, if you should have been so fortunate as to be an apothecary's apprentice, or book-keeper for a doctor, and have thus acquired the knowledge that will enable you to frame some compositions for yourself; in either case, apply to the patent office, and swear upon your *voire dire*, that you do *verily believe, that you are the true inventor or discoverer* of such compounds; whereupon a patent will issue, authorizing you to sell out for the 'benefit of the afflicted,' that of which the president knows about as much as Charles I. knew or possessed of the American continent, when he granted letters patent to Mr. Saybrook, for a slip of country from Long Island Sound to the Pacific ocean.

But let us turn from the *patentees* to the *patentors*; and inquire by what authority they grant letters for the cure of diseases.

By the statute of 1793, it is provided that—

"When any person or persons, being a citizen or citizens of the United States, shall allege that he or they have invented any new and useful art, machine, manufacture, or composition of matter, or any new and useful improvement on any art, machine, manufacture, or composition of matter, not known or used before the application, and shall present a petition to the secretary of state signifying a desire of obtaining an exclusive property in the same, and praying that a patent may be granted therefor, it shall and may be lawful for the said secretary of state to cause letters patent to be made out, in the name of the United States, bearing teste by the president of the United States."—*Ingersoll's Digest.* p. 656.

Now not one word is here said by the law, about drugs or medicines, or cures, or quack doctors; and we seriously doubt, whether the framers of the law ever contemplated an extension of its exclusive privileges, to the preparation and administration of remedies for the sick. The phrase 'composition of matter' evidently refers to the arts. Such obviously is and should be its meaning, for what can be more preposterous than a patent method of curing diseases; when not one of the whole catalogue, can be cured by the exhibition of any single medicine. A man would be entitled to the 'copy right,' a species of patent, for a chart of the Mississippi, by which a steam boat might be safely run from New Orleans to Louisville; but not a patent right for managing the boat on such a voyage. So, a chemist would be entitled to a patent for the discovery and preperation of Epsom salt or the sulphate of quina; but not for its exclusive application to the cure of diseases. If a man should discover the existence of aloes and rhubarb and ipecac, in plants which has not hitherto afforded them, he might fairly claim from the government the exclusive right to extract them from such plants. But these substances, by varying their proportions, may be formed into a hundred different kinds of pills, adapted in judicious hands, to a hundred different states of morbid action, though not *necessarily* a cure for any. How preposterous, then, to grant letters patent, for one of a series of compositions, that might be multiplied *ad infinitum!* But the patenting system, in its practical operation does not stop at this. A single composition will not do. This could not be offered as an infallible cure, for more than some twenty or thirty incurable maladies. Let the patents then be multiplied to 'No. 6' or upwards; print your directions accordingly, and let the retailers visit the sick daily and drench them *secundum artem, novem vel nigrum*: Thus under the *federal* patent you come to practise physic, the state laws to the contrary notwithstanding. Such is the origin of the Thomsonian or steam quackeries, which are the reigning epidemic of the day. That all who thus occupy themselves, do it in violation of the laws regulating the practice of medicine, and are obnoxious to their penalties, we have not a single doubt; but when our judges and legislators turn 'steam doctors,' and leave off butchering the constitution of the state, to butcher the constitutions of their fellow citizens, it would be credulous in the extreme, to believe that the statute against quackery is any thing more than a dead letter.

But it is time to enter on the review of the important works prefixed to the head of this article.

Of these books, the first is by *doctor* Thomson, who has favored the public—(that is such part of them as have the good sense to pay twenty dollars for a patent right) with an engraved likeness of himself, which we commend to the amateurs. The first 30 pages are composed of interlarded sketches, of the author's birth, labours and persecutions; of warnings to the good people against the 'regular faculty,' and of his system of physiology, pathology and therapeuticks. As most of the people are no doubt ignorant on all these important points, we shall make such extracts as the occasion seems to require.

"There are three things which have in a greater or less degree, called the attention of men, viz: Religion, Government, and Medicine. In ages past, these things were thought by millions to belong to three classes of men, Priests, Lawyers and Physicians. The Priests held the things of religion in their own hands, and brought the people to their terms; kept the scriptures in the dead languages, so that the common people could not read them. Those days of darkness are done away; the scriptures are translated into our own language, and each one is taught to read for himself. Government was once considered as belonging to a few, who thought themselves "born only to rule." The common people have now become acquainted with the great secret of government; and know that "all men are born free and equal," and that magistrates are put in authority, or out by the voice of the people, who choose them for their public servants.

While these, and many other things are brought where "common people" can understand them; the knowledge and use of medicine, is in a great measure concealed in a dead language, and a sick man is often obliged to risk his life, where he would not risk a dollar; and should the apothecary or his apprentice make a mistake, the sick man cannot correct it, and thus is exposed to receive an instrument of death, instead of that which would restore him to health had he known good medicine.

"It may be alleged, (said Dr. Buchanan,) that laying medicine more open to mankind, would lessen their faith in it. This indeed would be the case with regard to some; but it would have a quite contrary effect upon others. I know many people have the utmost dread and horror of every thing prescribed by a physician, who will nevertheless, very readily take a medicine which they know, and whose qualities they are in some measure acquainted with."

"Nothing ever can, or will inspire mankind with an absolute confidence in physicians, but by their being open, frank, and undisguised in their behaviour."

"The most effectual way to destroy quackery in any art or science, is to diffuse the knowledge of it among mankind. Did physicians write their prescriptions in the common language of the country, and explain their intentions to the patient, as far as he could understand them, it would enable them to know when the medicine had the desired effect; would inspire him with absolute confidence in the physician; and would make him dread and detest every man who pretended to cram a secret medicine or poison down his throat."

It is true that much of what is at this day called medicine, is deadly poison; and were people to know what is offered them of this kind, they would absolutely refuse ever to receive it as a medicine. This I have long seen and known to be true; and have labored hard for many years to convince them of the evils that attend such a mode of procedure with the sick; and have turned my attention to those medicines that grow in our own country, which the God of nature has prepared for the benefit of mankind. Long has a general medicine been sought for, and I am confident I have found such as are universally applicable in all cases of disease, and which may be used with safety and success, in the hands of the people.

After thirty years study and repeated successful trials of the medicinal vegetables of our country, in all the diseases incident to our climate; I can, with well grounded assurance, recommend my system of practice and medicines to the public, as salutary and efficacious."

It must be a matter of national pride, that if Germany produced a Luther and England a Bacon, America has sent up from its humblest walks, a self-taught and gifted Thomson, who has done for medicine what those eminent men achieved for religion and philosophy. But let us proceed:

"Possessing a body like other men I was led to enquire into the nature of the component parts of what man is made. I found him composed of the four elements—Earth, Water, Air and Fire. The earth and water I found were the solids; the air and fire the fluids. The two first I found to be the component parts; the two last kept him in motion. Heat, I found was life; and Cold, death. Each one who examines into it will find that all constitutions are alike. I shall now describe the fuel which continues the fire, or life of man. This is contained in two things—food and medicines; which are in harmony with each other; often grow in the same field to be used by the same people. People who are capable of raising their food and preparing the same, may as easily learn to collect and prepare all their medicines and administer the same, when it is needed. Our life depends on heat; food is the fuel that kindles and continues that heat. The digestive powers being correct, causes the food to consume; this continues the warmth of the body, by continually supporting the fire.

The stomach is the deposit from which the whole body is supported. The heat is maintained in the stomach by consuming the food; and all the body and limbs receive their proportion of nourishment and heat from that source, as the whole room is warmed by the fire which is consumed in the fire place. The greater the quantity of wood consumed in the fire place, the greater the heat in the room. So in the body; the more food, well digested, the more heat and support through the whole man. By constantly receiving food into the stomach, which is sometimes not suitable for the best nourishment, the stomach becomes foul, so that the food is not well digested. This causes the body to lose its heat—then the appetite fails; the bones ache and the man is sick in every part of the whole frame."

On these momentous speculations we shall not hazard a single remark. Still less shall we risk a comment on the noble effusion of original genius which followeth:—

"It has been acknowledged, even by those who are unfriendly to me and my practice, that my medicine may be good in some particular cases, but not in all. But this is an error. For there are but two great principles in the constitution of things, whether applied to the mind or body; the principle of life and the principle of death. That which contains the principle of life can never be tortured into an administration of death. If then a medicine is good in any case, it is because it is agreeable to nature, or this principle of life, the very opposite of disease. If it is agreeable in one case, it must be absolutely so in all."

The following extract will no doubt impart much consolation and quiet of conscience, to some of the regular faculty. It would be ill-natured to assign the reason:

"It is true that the study of anatomy, or structure of the human body and of the whole animal economy, is pleasing and useful; nor is there any objeetion to this, however minute and critical, if it is not to the neglect of first great principles, and the weightier matters of knowledge. But it is no more necessary to mankind at large, to qualify them to administer relief from pain and sickness, than to a cook in preparing food to satisfy hunger and nourishing the body. There is one general cause of hunger and one general supply of food; one general cause of disease and one general remedy. One can be satisfied, and the other removed, by an infinite variety of articles, best adapted to those different purposes—That medicine, therefore, that will open obstruction, promote perspiration, and restore digestion, is suited to every patient, whatever form the disease assumes, and is universally applicable. And acute disorders, such as fevers, cholics, and dysentary, may be relieved thereby, in twenty-four or forty-eight hours, at most."

But we must come to particular diseases:

"No person ever yet died of a fever! for as death approaches, the patient grows cold, until in death, the last spark of heat is extinguished. This, the learned doctors cannot deny; and as this is true, they ought, in justice, to acknowledge that their whole train of depletive remedies, such as bleeding, blistering, physicing, starving, with all their refrigeratives; their opium, mercury, arsenic, antimony, nitre, &c. are so many deadly engines, combined with the disease, against the constitution and life of the patient. If cold, which is the commonly received opinion, (and which is true,) is the cause of fever, to repeatedly bleed the patient and administer mercury, opium, nitre, and other refrigerents to restore him to health, is as though a man should, to increase a fire in his room, throw a part of it out of the house, and to increase the remainder, put on water, snow and ice?"

The figurative style of the next paragraph must excite a general regret, that our author had not taken to the trade of poetry instead of medicine:

"Having described the two kinds of fever which are the most alarming, they being most fatal, I shall pass over those of a less alarming nature, and merely observe, that there is no other difference in all cases of fever, than what is caused by the different degrees of cold, or loss of inward heat, which are two adverse parties in one body, contending for power. If the heat gains the victory, the cold will be disinherited and health will be restored: but, on the other hand, if cold gains the ascendancy, heat will be dispossessed of its empire, and death will follow of course. As soon as life ceases, the body becomes cold, which is conclusive evidence that its gaining the victory is the cause of death. When the power of cold is nearly equal to that of heat the fever or strife between the two parties, may continue for a longer or shorter time, according to circumstances; this is what is called a longer fever, or fever and ague. The battle between cold and heat will take place periodically, sometimes every day, at other times, every other day, and they will leave off about equal, heat keeping a little the upper hand."

Again:—

"Much has been said by the doctors concerning the turn of a fever, and how long a time it will run. When it is said that a fever will turn at such a time, I presume it must mean that it has been gone; this is true, for it is then gone on the outside, and is trying to turn again and go inside, where it belongs. Instead of following the dictates of nature and aiding it to subdue the cold, the doctor uses all his skill to kill the fever. How, would I ask, in the name of common sense, can any thing turn when killed? Support the fever and it will return inside; the cold, which is the cause of disease, will be driven out, and health will be restored. In all cases called fever, the cause is the same in a greater or less degree, and may be relieved by one general remedy. The cold causes canker, and before the canker is seated the strife will take place between cold and heat; and while the hot flashes and cold chills remain, it is evidence that the canker is not settled, and the hot medicine alone occasionally assisted by steam, will throw it off; but as the contest ceases, the heat is steady on the outside; then canker assumes the power inside; this is called a settled fever. The truth is, the canker is fixed on the inside and will ripen and come off in a short time, if the fever is kept up so as to overpower the cold. This idea is new and never was known until my discovery. By raising the fever with Nos. 1 and 2, and taking off the canker with No. 3, and the same given by injections, we may turn a fever when we please; but if this is not understood, the canker will ripen and come off itself, when the fever will turn and go inside and the cold will be driven out; therefore they will do much better without a doctor than with. The higher the fever runs, the sooner the cold will be subdued; and if you contend against the heat, the longer will be the run of the fever, and when killed, death follows.

When a patient is bled, it lessens the heat and gives double power to the cold; like taking out of one side of the scale and putting it in the other, which doubles the weight, and turns the scale in favor of the disease. By giving opium it deadens the feelings; the small doses of nitre and calomel tend to destroy what heat remains, and plants new crops of canker, which will stand in different stages in the body, the same as corn planted in the field every week, will keep some in all stages; so is the different degrees in canker. This is the reason why there are so many different fevers as are named; when one fever turns another sets in and so continues one after another until the harvest is all ripe, if the season is long enough: if not, the cold and frost takes them off—then it is said they died of a fever. It might with as much propriety be said that the corn killed with frost, died with the heat. The question, whether the heat or cold killed the patient, is easily decided, for that power which bears rule in the body after death is what killed the patient, which is cold—as much as that which bears rule when he is alive is heat. When a person is taken sick, it is common to say, I have got a cold and am afraid I am going to have a fever; but no fears are expressed of the cold he has taken; neither is it mentioned when the cold left him. The fashionable practice is to fight the remains of heat till the patient dies, by giving cold the victory; in which case, is it not a fact that the doctor assists the cold to kill the patient? Would it not have been more reasonable, or likely to have cured them, when the fever arose to throw off the cold, to have helped the fever and give nature the victory over its enemy, when the health would be restored the same as before they took the cold."

Against this acute and edifying extract, we should hardly be so imprudent as to run a tilt. Perhaps *doctor* Potter or *doctor* Swaim may take the subject up.

Orator Robinson informs us, that *doctor* Thomson's system made a very favorable impression on the mind of Dr. Rush. Now we wonder what that eminent advocate of the lancet must have thought of the following:—

"The practice of bleeding for the purpose of curing disease, I consider most unnatural and injurious. Nature never furnishes the body with more blood than is necessary for the maintenance of health; to take away part of the blood, therefore, is taking away just so much of their life, and is as contrary to nature, as it would be to cut away part of their flesh. Many experiments have been tried by the use of the lancet in fevers: but I believe it will be allowed by all, that most of them have proved fatal; and several eminent physicians have died in consequence of trying the experiment on themselves. If the system is diseased, the blood becomes as much diseased as any other part; remove the cause of the disorder and the blood will recover and become healthy as soon as any other part; but how taking part of it away can help to cure what remains, can never be reconciled with common sense."

The following paragraph must be a sore application to our friend Dr. Daniell, of Savannah, who proposes to cure almost all fevers by sinapisms:—

"There is no practice used by the physicians that I consider more inconsistent with common sense, and at the same time more inhuman, than blistering to remove disease; particularly insane persons or what the doctors call dropsy on the brain; in which cases, they shave the head and draw a blister on it.—Very few patients if any ever survive, this application. What would be thought if a scald should be caused by boiling water to remove disease? Yet there is no difference between this and a blister made by flies. I have witnessed many instances where great distress and very bad effects have been caused by the use of blisters; and believe I can truly say, that I never knew any benefit derived from their use. It very frequently causes stranguary, when the attempted remedy becomes much worse than the disease."

But it is time to enter the steam bath. Here the *doctor* is quite at home; and when at home does least harm to his patients.

"Steaming is a very important branch of my system of practice, which would in many cases without it, be insufficient to effect a cure. It is of great importance in many cases considered by the medical faculty as desperate; and they would be so under my mode of treatment, if it was not for this manner of applying heat to the body, for the purpose of reanimating the system and aiding nature in restoring health. I had but little knowledge of medicine, when through necessity, I discovered the use of steaming, to add heat or life to the decaying spark; and with it I was enabled, by administering such vegetable preparations as I then had a knowledge of, to effect a cure in cases where the regular practitioners had given them over.

The method adopted by me, and which has always answered the desired object, is as follows--Take several stones of different sizes and put them in the fire till red hot, then take the smallest first, and put one of them into a pan or kettle of hot water, with the stone about half immersed—the patient must be undressed and a blanket put around him so as to shield his whole body from the air, and then place him over the steam. Change the stones as often as they grow cool, so as to keep up a lively steam, and keep them over it; if they are faint, throw a little cold water on the face and stomach, which will let down the outward heat and restore the strength—after they have been over the steam long enough, which will generally be about 15 or 20 minutes, they must be washed all over with cold water or spirits and be put in bed, or may be dressed, as the circumstances of the case shall permit."

The *originality* of this method certainly entitled it to a patent.

The next 40 pages of our author, are devoted to the natural and pharmaceutic history of his materia medica. His hopes rest upon *lobelia inflata*, *cayenne pepper*, *ginger*, *black pep-*

*per, bayberry, white pond lilly, the hemlock tree, rosemary, sumach, witch hazle, raspberry, squaw weed, balmony, pop ar bark, barberry, bitter root, goldenscal, peach meats, cherry stones, myrrh, spirit of turpentine, camphor, and lady's slipper;* but he has, condescendingly, added the names of some thirty or forty more, among which we observe *elecampane, yellow dock, summersarosy,* and other *new* discoveries.

Now come the 'preparations and compositions.' These run through Nos. 1, 2, 3, 4, 5, & 6, besides 'nerve powder,' 'cough powder,' and 'vegetable powder,' with plasters and ointments.

'No. 1.' Comprehends the preparations of lobelia—'No. 2.' of cayenne pepper—'No. 3.' of bayberry, pond lily and hemlock—'No. 4.' of the bitters—'No. 5.' of poplar bark, bayberry, peach meats and cherry stones—'No. 6.' (rheumatic drops) of myrrh, cayenne and brandy. Nerve powder is composed—but we really beg to be excused from prosecuting this branch of the subject any further; and shall wind up with the following momentous information to our patients generally.

"STOCK OF MEDICINE FOR A FAMILY.

1 oz. of the Emetic Herb,
2 ozz. of Cayenne,
1-2 lb. Bayberry root bark, in powder,
1 lb. of Poplar bark,
1 lb. of Ginger,
1 pint of the Rheumatic Drops.

"This stock will be sufficient for a family for one year, and with such articles as they can easily procure themselves when wanted, will enable them to cure any disease, which a family of common size may be afflicted with during that time. The expense will be small, and much better than to employ a doctor and have his extravagant bill to pay.

In immediate connexion with the *doctor's* medicine chest, we shall place his directions for what he calls a regular course of medicine, which is the *new method* of curing *all* diseases.

"As I have frequently mentioned a regular course of medicine, I will here state what is meant by it, and the most proper way in which it is performed. Firstly, give No. 2 and 3, or composition, adding a tea-spoonful of No, 6; then steam, and when in bed repeat it, adding No. 1, which will cleanse the stomach and assist in keeping up a perspiration; when this has done operating, give an injection made with the same articles. Where there are symptoms of nervous affection, or spasms, put half a tea-spoonful of the nerve powder into each dose given, and into the injection. In violent cases, where immediate relief is needed, Nos. 1, 2, 3, and 6, may be given together. Injections may be administered at all times and in all cases of disease to advantage; it can never do harm, and in many cases, they are indispensably necessary, especially where there is canker and inflammation in the bowels, and there is danger of mortification, in which case, add a tea-spoonful of No. 6. In cases of this kind, the injection should be given first, or at the same time of giving the composition, or No. 3."

In this way the *doctor* proposes, as we have said, to cure nearly all diseases. Let us take a few examples from the back of his book:—

MEASLES.

"This disease is very common, especially among children, and is often attended with bad consequences, when not properly treated. It is a high state of canker and putrefaction; and if the determining powers are kept to the surface, it will make its appearance on the outside, and go off itself; but if cold overpowers the inward heat, so as to turn the determining powers inward, the disease will not make its appearance, and the patient will become much distressed, frequently producing fatal consequences, if some powerful stimulant is not administered to bring the disorder out. To give physic in cases of this kind is very dangerous, as it strengthens the power of cold and keeps the canker and putrefaction inside, which sometimes seats upon the lungs and causes consumption; or turns to the stomach and bowels, when they die suddenly, as has been the case with hundreds, for a few years past. I have attended a great many cases of the measles in the course of my practice, and never lost one; and never have known of any that have died of this disorder, who were attended by any of my agents. When the symptoms make their appearance, give a dose of the composition powder, or of No. 2; then give the tea of No. 3, to guard against canker, and add some No. 2, to overpower the cold; and when the second dose is given, add No. 1, to clear the stomach and promote perspiration; as soon as this takes place, the disorder will show itself on the outside. By continuing to keep the determining power to the surface, nature will take its regular course, and the disease will go off without injuring the constitution. If the bowels appear to be disordered, give an injection; and be careful to keep the patient warm."

SMALL POX.

"This disease is the highest state of canker and putrefaction, which the human body is capable of receiving, and is the most contagious, being taken in with the breath, or may be communicated by inoculation, in which case it is not so violent and dangerous as when taken in the natural way. The distressing and often fatal consequences that have happened in cases of the small pox, are more owing to the manner in which it has been treated than to the disease. The fashionable mode of treatment in this disease has been to give physic, and reduce the strength, by starving the patient and keeping them cold. This is contrary to common sense, as it weakens the friend and strengthens the enemy; and the same cause would produce similar effects in any other disorder. All that is necessary is to assist nature to drive out the canker and putrefaction, which is the cause of the disease, by keeping the determining powers to the surface, in which case there will be no danger. The same manner of treatment should be used in this complaint as has been directed for the measles. The canker-rash, and all kinds of disease that a person is not liable to have but once, such as chicken-pox, swine-pox, &c. are from the same cause and must be treated in a similar manner."

CONSUMPTION.

"This complaint is generally caused by some acute disorder not being removed, and the patient being run down by the fashionable practice, until nature makes a compromise with disease, and the house becomes divided against itself. There is a constant warfare kept up between the inward heat and cold, the flesh waters away in consequence of not digesting the food, the canker becomes seated on the stomach and bowels, and then takes hold of the lungs. When they get into this situation it is called a seated consumption, and is pronounced by the doctors to be incurable. I have had a great many cases of this kind and have in all of them, where there was life enough left to build upon, been able to effect a cure by my system of practice. The most important thing is to raise the inward heat and get a perspiration, clear the system of canker, and restore the digestive powers, so that food will nourish the body and keep up that heat on which life depends. This must be done by the regular course of medicine, as has been directed in all violent attacks of disease, and persevering in it till the cause is removed.

This complaint is called by the doctors a hectic fever, because they are subject to cold chills and hot flashes on the surface; but this is an error, for there is no fever about it; and this is the greatest difficulty—if there was it would have a crisis and nature would be able to drive out the cold and effect a cure; the only difficulty is to raise a fever, which must be done by such medicine as will raise and hold the inward heat till nature has the complete command, When the patient is very weak and low, they will have what is called cold sweats; the cause of this is not understood; the water that collects on the skin does not come through the pores, but it is attracted from the air in the room, which is warmer than the body, and condenses on the surface; the same may be seen on the outside of a mug or tumbler on a hot day, when filled with cold water, which is from the same cause. It is of more importance to attend to the preventing of this complaint than to cure it. If people would make use of those means which I have recommended, and cure themselves of disease in its first stages, and avoid all poisonous drugs, there would never be a case of consumption or any other chronic disorder."

We owe an apology perhaps to the readers of the Western Journal, for so an extended a notice of the '*New Guide to Health.*' So long as its circulation was confined to the grossly ignorant, no journalist could be excused for noticing its thousand and one fooleries. But it is not at present limited to the vulgar. Respectable and intelligent mechanicks, legislative and judicial officers, both state and federal, barristers, ladies, ministers of the gospel, and even some of the medical profesion,

"Who hold the eel of science by the tail,"

have become its converts and puffers.

"The dog star rages and beyond a doubt
"All bedlam or parnassus is let out."

At such a critical stage of the disorder, it seems proper, that the faculty should know something of its causes, and this we have sought to communicate.

We shall for the present dismiss this artful demagogue and mischievous impostor, with the following reflections, in which every honorable and intelligent member of the profession, will concur.

1. His success in acquiring popularity, is in no small degree attributable to his railings against the educated physicians.

2. By presenting what he calls a system of principles, to those who cannot perceive their absurdity, he acquires their confidence.

3. Whatever may be found of physiology or pathology in his book, has been picked up from the popular works on medicine.

4. His remedies consist of plants long known to the profession; many of them now in use, and others rejected as inert after repeated trials. Even his boasted lobelia has been known and used as a medicine, by the physicians for 30 years.

5. The steam bath with the subsequent application of cold water, has been used in all ages and among all people. It is well known to be a favorite remedy with our North American Indians, from whom Thomson adopted his manner of applying it. Of all his remedies this is perhaps at once the most harmless and the most efficacious.

6. Most of the medicines on which he relies are active stimulants and may, therefore, cure the diseases to which that class of remedies are adapted; while they produce the most fatal effects, in maladies of an inflammatory character, for which they are invariably used. Of this several examples have fallen under our own observation, and cases have been communicated to us from all quarters. Herein lies the abuse, which calls for the interposition of the faculty. If their friends and patients, while the delusion rages, will not listen to what they say, it is not the less their duty to speak. The consciousness of having rescued but a single credulous victim from his impending fate, should, with a physician of humanity, outweigh a thousand ungenerous insinuations against the motives which governed him.

8. There is nothing original, in the union of emetics with sweating and heating medicines. From time immemorial, taking a vomit and then a composing draught, drinking hot tea, and bathing the feet in hot water, so as to take a good sweat, is a practice that has prevailed in the profession, and among the people. It was reserved for one *Samuel Thomson* to take out a patent, for a clumsy and often dangerous method, of arriving at the *very same result*; and thus to become, in the eyes of the weak-minded and superstitious, one of the greatest geniuses and benefactors of the human race! Why does not some one get out a patent, for preventing the small pox by vaccinating on the nose, instead of the arm? It might be averred, that it would not, *then*, be necessary to repeat the operation every seven years, or oftener if a man moved from one town to another. Such vaccinations might be as effectual as those in the arm, and could be referred to and published—not as testimony in favour of the *Cow-pock*, but in favour of inserting it in the 'end of the snout.' Thus in a short time we should find half the community—(we mean half in point of numbers,) turning up their noses at their incredulous and bigotted brethren, who might prefer to have the carbuncle on their arms.

7. Intelligent and candid men, not of the medical profession, ought to be apprized, that the strong medicines are not confined to the mineral kingdom; but that the greatest poisons are of vegetable origin, and the number not a few. Even prussic acid, the deadliest poison ever discovered, may be distilled from *doctor* Thomson's *peach meats* and *cherry stones*.

9. Lastly, we might apply to the whole Thomsonian farrago of doctrines and recipies, the remark which Blumenbach is said to have made upon phrenology—'That which is true is not new; and that which is new is not true.'

Such being the character of the *system* under review, we cannot forbear to express our surprise and sorrow, that it should have found a public advocate in the reverend author of the *Fifteen Lectures*. That a clergyman of reputed piety, learning, genius and eloquence, could be found to lend himself to the propagation of such stupid and dangerous quackeries, originating with one of the most illiterate and indolent men in society, could not have been anticipated: That he would labor to hide their deformities behind the veil of his polished elocution, is a prostitution of his talents: That he could stand up and decry a profession, which has long been closely associated with his own; declaim against *regular* studies; and level the battering ram of his wit against a superstructure, erected by ages of toil in the dearest interests of humanity, does little credit to his feelings: That he would ransack encyclopædias and digests, to acquire a smattering of medical theories, and artfully interweave them with the nonsense of a ridiculous, but mischievous empyricism, gives but poor indication of his rectitude: That he should garnish the whole with texts of scripture, and attempt to give to heresy in medicine, a dangerous passport to the confidence of the religious community, by blending it with sentiments of devotion, argues still less for his piety:—and yet all this and more has been done by the reverend, author before us.

Let us look at a few specimens:—

"Animal life, as it operates on the human body in health and in disease, has been considered the primary and grand object of the attention of the physician. And some of its most oovious properties are sensibility, irritability, and excitability. These are the *effects* of vitality, which have been mistaken for *vitality* itself.

*Some* physicians have supposed that the vital principle may lie dormant in a quiescent state, like *latent* heat, and afterwards be made to shew itself, like heat, by the application of stimuli. But the reasoning is fallacious; it is merely analogical—drawn from a material subject, *heat*, to prove the phenomena of an immaterial subject—the *spirit* of life. It would be better reason, to attempt to prove that the spirit is *latent*, when the body is dead, because we cannot perceive its effects; than to attempt to establish from latent *heat*, a latent state of mind. For if in fainting, or catalepsy, it can be established that the spirit is merely latent--it may as well be latent in the grave to the day of judgment: for in the argument respecting an immaterial *substance*, whose very essential quality is *activity*—and without which it could not be—the *latency* of *one* hour, or *one hundred thousand millions*, could not at all change the conditions of the question; nor relieve the disputant from the direful consequences of making the soul a material *substance*.

I know some physicians distinguish between the rational soul and the vital principle of animal life. But the distinction is, perhaps, not clearly understood. There is in animals something far superior to mere vitality. A *plant* has *vitality*—its *life* and *death*. And Dr. Brown's theory was applied, with great success, to plants; and supported them with superior energy and vigor, in the high latitudes of Scotland! But in animals, besides vitality, we perceive *thought*, *reason*, *memory*, *design*, and *perseverance*, with a great number of the noble passions which animate man--*love*, *gratitude*, *affection*, *friendship*, *grief* and *bitter woe*, even to the destruction of life.

A very eminent and pious philosopher, considered these phenomena, as the operation and agency of God, moving and directing his own universe to the final *issue* and grand results of the eternal Judgment. This, by the way, is a very old opinion, and has been beautifully embodied by the poet, in these celebrated lines:

"All are but parts of one stupendous whole,
Whose body, *nature* is, and God the soul:
That, chang'd through all, and yet in all the same,
Great in the earth as in the etherial frame;
Lives in the sun, refreshes in the breeze,
Glows in the stars, and blossoms in the trees;

Lives through all *life*, extends through all extent,
Spreads undivided, operates unspent;
Breathes in our soul, informs our mortal part,
As full, as perfect, in a hair as heart;
As full, as perfect, in vile man that mourns,
As the rapt seraph that adores and burns.
To him no high, no low, no great, no small;
He fills, he bounds, connects, and equals all!"

This is not the doctrine of SPINOZA, who made God the *soul* of the world; but the pious doctrine of a universal providence, and the omnipresence of the Deity in the government of the world. Look at the smallest plant or insect, you behold him there, in his matchless wisdom and sustaining power—forming the mechanism and moving the vitality of a creature so small and inconsiderable, and apparently worthless in the great sum of things. The Psalmist took a most striking and comprehensive view of this sublime and glorious theme. "Whither shall I go from thy spirit? Or whither shall I flee from thy presence? If I ascend up into heaven, thou art there! If I make my bed in hell, behold thou art there!! If I take the wings of the morning, and dwell in the uttermost parts of the sea, even there shall thy hand lead me, and thy right hand shall hold me. If I say surely the darkness shall cover me, even the *night* shall be light round about me!"

This was the true sentiment and doctrine of the ancient philosophers—the presence and superintendance of the Deity every where. They were not Atheists—although the miserable SPINOZA wrested their doctrine to his own malignant and deadly purpose. But he might well do that, when he turned the Jewish Scripture to the same account—for he was a Jew, and deeply read in the old testament. But the *wasp* can extract poison from the flower: So did his perverted *soul* draw death from the wells of salvation!

As the doctrine of *life* and *health* cannot be known by reasoning *a priori;* but must be deduced from experience and observation, some very eminent men have thought that its laws and principles should be divided in a different manner from that of the scholastic mode: That so many divisions of the theory of life and disease, which have prevailed since the days of GALEN, have not only embarrassed but bewildered the subject; and that the laws and principles, therefore, should be divided in a different manner—1st, that the human blood is the recipient and vehicle of heat and life to the several parts; 2d, from many experiments, *pure air* appears to be the pabulum of irritability; for the absence of pure air destroys life sooner than the defect of any other natural *substance*; 3d, the next in importance to the animal economy, seems to be the nervous fluid, or the medulla of the brain and spinal marrow; for they have all the same nature and origin; 4th; sensibility, residing in the organ of sense, connecting the mind with the external world.

All this is very *fine*, but has still less affinity to the proposition, that all diseases are curable by steam, indian tobacco, brandy and 'No. 6,' than to the *intimation* that Thomson was commissioned by Heaven, to give a revelation to man on the effects of those renowned agents. That our *reverend* professor, at least does not discourage the idea, that his *magnus*

*apollo* was thus sent, may be collected from the following extracts:—

"Dr. THOMSON had this opinion from the effects he himself had seen; and his Narrative is convincing from its very form and features. He tells us he was *illiterate*, and he was poor; oppressed by a young, helpless, and sickly family; the practice pursued did not agree with their constitutions, nor diseases; he was, from nature, inclined to try the virtue and operation of plants; the gift of healing, it was impressed upon his mind, God had given to him; necessity, when his child was dying, forced him to try; he was successful; success encouraged him to go on; his neighbors applied to him in the hour of calamity; he relieved their complaints, his time was consumed, his reward *nothing;* he consulted with his wife and friends, whether he should abandon the practice, or abandon his farm and yield to these pursuits; he was counselled to follow his own inclination. Still believing he had a call from Providence, and a degree from the God of nature, he commenced, in form, the healing art. His cause and claims are before the world; laid before the Government of his country; his remedies submitted to the experience of scientific men, and eminent physicians; tried by a jury of his country for his cures, and even perjury could not substantiate a plea against him! This is something very different from all the pretensions to the healing art ever yet set up in the world.

"This new practice has this *vast and high prerogative, it cannot be wrong, and will not kill; no mistakes are fatal here;* no unexpected and sudden death when you think the patient is just about to do well. I know a physician who put his patient through a course of mercury; in the evening he said he was doing well—he called in the morning, and inquired for his patient, and was informed he was dead! He was struck dumb!—looked on the lifeless corpse, and departed without uttering a single word, with a load of wo upon his heart, that I would not have suffered for a mountain of gold! Yet he could not be blamed; he practised according to his education, and was utterly deceived in the operation of his medicines. He thought they were curing the patient; but alas! they were digging his grave!

The power of prejudice and the empire of pride, may prevail for a season; but the soul will at last arise and re-assert the majesty of her own nature, and shew unto the world, that "there are gifts, beyond the power of education and knowledge, which learning cannot bestow." Learning will neither make a *great* man, nor a *great* physician, but it will highly advance the usefulness of those who are great by nature; who have received the patent of their dignity from God Almighty. Dr. Waterhouse said of Dr. Thompson, he had taken a degree from the school of nature—a diploma from her unerring hands. The very course of that education to which Dr. Waterhouse has so handsomely alluded, was calculated to instruct the author of the new system in useful remedies, and deliver his mind from every bias but the force of experience and truth. With a mind entirely uninfluenced by all authority, unmoved and unobstructed by any thing which had gone before him, he possessed an advantage which, I am persuaded, none ever possessed who were educated in the schools—where we are introduced to the fellowship of *wisdom* by the authority of books and professors.

"It is impossible for the most independent mind to perfectly retain its freedom; it will insensibly bow to the opinions of some celebrated or splendid authority. In after life, indeed, and by much experience, some superior souls are enabled to cast off the shackles of education; but they are the fewest number of that mighty host, which walk forth from the schools of the world, to propagate the errors of their predecessors. Dr. THOMSON had nothing of all this to encounter; he was led by the hand of nature; and without being aware of the fact, he was travelling in the path of the Indian, the German and Celtic doctors---the doctors of antiquity, who without complaint or failure, practised on the unnumbered millions, who overturned the empire of the Romans; and still practice on all the nations of the Gentile world. He is, therefore, now a professor in the most ancient and extensive medical school of the world. A school, not on the decline and about to perish--but one beginning to revive—to put on strength—to extend her conquests, until the learned and the unlearned shall be gathered under the shadow of her wings, and triumph in the splendor of her acquisitions. And we see the dawn of this glorious era, which shall transform the face of the world."

To these flowing paragraphs we shall add from *doctor* Thomson, for the benefit of 'all whom it may concern,' the following important recipe—

"TO MAKE CHICKEN BROTH.

"Take a chicken and cut it in pieces; put the gizzard in with it, opened and cleaned, but not peeled. Boil it till the meat drops from the bone. Begin to give the broth as soon as there is any strength in it; and when boiled eat some of the meat. Let it be well seasoned. This may be given instead of the milk porridge, and is very good for weak patients, particularly in cases of the dysentery.

"When the operation of medicine is gone through, I have said that the patient may eat any kind of nourishing food his appetite should crave; but the best thing is, to take a slice of salt pork boiled, or beef-steak, well done, and eat it with pepper sauce; or take cayenne, vinegar, and salt, mixed together, and eat with it, which is very good to create an appetite and assist the digesture."

Recurring to 'orator' Robinson, we shall, for the present, dismiss both him and his coadjutors, with the closing sentence of his last lecture, and another extract from the DUNCIAD:—

"And let it be remembered, if this system of practice is true, it will have the peculiar blessing of the Almighty upon its side; because it brings the power, the benefits, and the beneficial results of a safe medicine, within the reach of the poor; into their dear *distressed* families, who often perish for the lack of the means to procure medical aid! This single *benefit* cannot fail of drawing down from heaven the peculiar blessing of Him, who bowed his majesty and left his throne, and veiled his glories, to enter the world, and preach "the gospel to the poor!"

"Imbrown'd with native bronze, lo! Henley stands
"Tuning his voice, and balancing his hands.
"How fluent nonsense trickles from his tongue!
"How sweet the periods, neither said, nor sung!

"Note.—J. Henley the Orator: he preached on the Sundays on Theological matters, and on the Wednesdays, upon all other sciences. Each auditor paid one shilling. He declaimed some years against the greatest persons."

"This man had a hundred pounds a year given him for the secret service of a weekly paper of unintelligible nonsense, called the Hyp-Doctor."

It appears from the Lectures, that in the state of New-York the *steamers* and the *steamed*, have combined, in petitioning the legislature for permission to carry on the trade. In Ohio a similar memorial was presented to the last session of the general assembly, and referred to the committee on the *Penitentiary*. A notice so flattering, has encouraged the 'gifted' to renew their applications, and that our journal may not be charged with indifference to their interests, we shall insert an exact copy of one of them, lately transmitted to us by a respectable correspondent, who informs us, that it was handed, by the author, to a member of the legislature, to be laid before that honorable body, by the Governor:

"Sir Honorable and respectable Alen Trimble Governor of the State of Ohio I envocate my earnest prayers to you and your predesesors in office to Jere McClain Sec. And vice president and state prothonotry praying to giving you a minute introduction of my dolorous situation and awful calamities difficulties dangers and distructions that I have traversed and still hangs over my head clouds of danger and darkness unless I can corespond to your feelings the necessity of your great and important assistance to my deseres requests colls and prayers praying that you would consider my inexpresable necessities and grant to me Doc. Uri Martin of Morgan county Ohio a licence to lawfully authorize me to practice medicines of every System agreeable to the Laws of Ohio And conscientiously discharge my duty in every actuating Sphere of Allusion in *alphabetical* or *syntactical* or *electrical* or *physical* or *mental* or *surgical* or *astronomical* or *plisiognomical* or *medical* Terms or words or sentiments or sentences or physical and surgical modes customs rudiments and laws that when I am contiously safe that I may be lawfully safe in adminestering any medicenes or using any means to restore them that is deficient in health agreeable to the dictates of my Judgment and concience and agreeable to my titled systamatical stile and agreeable to my epithetical affirmed advertised notification to the State house door Ohio and to the United States house door that I would devote my time and talents impartially to the practice of Physic in every shape under the well adapted systems and long attentive studies and satisfactory experience I Doc Uri Martin Morgan Co. have counseled with Dan Martin and Calvin Martin of the same county and William Granville Capt of Mead to Belmont co. & Will. McAma Gospel preacher of Liberty Licking Co. I pray that the law builders——of state of Ohio may licenc me with preveliged Cognized Diploma agreeable to the statutes of the United States of America and under this title Archeotybe Medical botonical Society."

Leaving the 'gifted' brotherhood, to work out the Millenium with 'No. 3' feather few, and clover heads, a 'CURE for CANCERS, SORE LIPS, and OLD SORES,' we shall proceed to a passing notice of a few more of the people's doctors; and first, of *doctor* Swaim--whose 'MEDICINE may without extravagance,' as he himself informs us, 'be considered as a *sacred* boon to the afflicted.' Deserving, however, as it may be of this *disinterested* commendation, it cannot supersede *doctor* Thomson's materia medica, as it is infallible in no more than the following meagre catalogue, of the evils which flesh is heir to:—

"Scrofula or King's Evil, Ulcerated Sore Throat, long standing Rheumatic Affections, Diseases of the Skin, White Swelling and Diseases of the Bones, and all cases generally of an Ulcerous character; Chronic and Nervous diseases arising in debilitated constitutions; but more especially for Syphilis, or affections arising therefrom, as Ulcers of the Larynx, Throat and Nose, Nodes, &c. and those evils occasioned by an improper *use of mercury*, &c. &c.—It has been found to be a most useful *spring* and *fall* alternative for debilitated and bilious persons; it is also beneficial in dyspeptic and nervous complaints, and most internal diseases where the lungs and chest are supposed to be affected," &c.

*Doctor* Swaim was, we believe, a sadler and harness maker, in one of the 'cow-paths' of the renowned and ancient city of 'New Amsterdam.' Such an occupation of course led him into the society of ostlers and farriers, from whom he seems to have imbibed the first notions of becoming a *doctor*. While this predisposition was upon him, fortunately for the world, he met with a recipe, for some sort of decoction, or 'diet drink, proposed by a French physician; and immediately invented a PANACEA; which, he vouchsafed to sell to the afflicted, for three dollars a bottle, with a suitable discount to wholesale purchasers! Recollecting that 'a prophet is not without honor save in his own country,' he was not slow in transporting both himself and his *elixir vitæ*, to the other side of the Jerseys; and opened shop in the city of rectangles and staid habits, where the powers of his decoction soon became so great, as to extort from the very *magnates* of the profession, those sententious little passports to the confidence of the people, which have ever since graced the first pages of his book. In the true spirit of ambition, he speedily sought for new worlds, and embarked his bottles on the ocean; but instead of steering, like his patients, for the 'hollow of the earth,' he wisely directed the prow of his lugger to the famous city of London. Its commander was Dr. William Price, whose first bulletin from Great Britain, was in these encouraging words, to wit:

*Liverpool, (England.)*

"The Vegetable Syrup, called Swaim's PANACEA, prepared by Mr. Swaim, of Philadelphia, has recently been introduced here by Dr. Price, from the United States of America, where it is now extensively used in the treatment of a variety of Chronic Diseases.

Of the efficacy of this preparation Dr. Price has had abundant and most satisfactory evidence, during a course of experiments made under his direction, whilst Surgeon of the Pennsylvania Hospital; and since his arrival in England, he has had the good fortune of witnessing many additional instances of its successful administration.

The diseases in which this medicine has been particularly useful, are those arising from constitutional causes—as in the various forms of Scrofula, whether affecting the bones, joints, or soft parts; and in cases where a disposition to this disease is manifested by debility only, it operates as a preventive to the local disease by its beneficial effects on the constitution. It is equally efficacious in *mercurial* disease, and in the secondary forms of syphilis; and has lately been given with marked success in chronic diseases of the liver, which had resisted the careful exhibition of mercury. It has, likewise, very recently been administered with decided advantage by one of the most distinguished Surgeons in London, in a case which had entirely destroyed the right eye of the patient, and a great portion of the side of the face.

WM. PRICE, M. D."

"With horns and trumpets now to madness swell,
"Now sink in sorrows with a tolling bell!"

Meanwhile *doctor* Swaim, with his 'barrels of decoctions,' was busy at home, and ere long—

"Men bearded, bald, cowl'd, uncowl'd, shod, unshod;
"Peel'd, patch'd, and pye bald, linsey woolsey brothers,
"Grave mummers! Sleeveless some and shirtless others"—

stood forth as its trumpeters; and testified to its great efficacy in curing the *mercurial* diseases, produced by the 'regular Doctors.' But in the midst of this splendid success—*lugubri dictu*—what should be discovered but *corrosive sublimate*, the STRONGEST of the mercurial preparations, in this very decoction of all the salutiferous plants! Where is now the *panacea? Sic transit gloria mundi.*

"Is it enough? or must I, while a thrill
"Lives in your sapient bosoms, cheat you still?"

So asks the Genius of Quackery, with an ungrateful smile of contempt; but the 'people' are not easily choked off. They wish for *more;* and why should they not have it? Is not their will the supreme law of the land? The 'riglar doctors' charge high, and poison them into the bargain with 'mercury, calomel, *bleeding*, *blisters*, arsenic, and digitalis.' So says Prof. Rafinesque, Ph. D., M. D. and Pulmist,' and the 'people' cry amen! Now, then, is the *golden* moment of opportunity, at least for the Professor, who charges for—

"First visit in any cases, $5; but every successive visit only $1. The Poor taught to use the Tan bark for $1. Liable individuals taught how to prevent the disease for $1."

Rafinesque is a name not unknown to our Backwoods readers; but many of them may ask, whether the Pulmist is the same with the fishtaker, and ancient chronicler of Kentucky? We answer, that such *appears* to be the fact; and dismal, indeed, must be the 'times,' that can make an apothecary's muller of such a learned head. We have not had the advantage of seeing the professor's 'doctor book,' the title of which is prefixed to this article, but his 'circular' lies before us, and affords intrinsic evidence, that the *pulmist* is no other than the distinguished *antiquary* who lately settled the limits, and *located* the wigwams, of our Indian tribes for the last three thousand years! Strange metamorphosis of genius! It was, doubtless, while inquiring into the arts and sciences of these savage hordes, long since extinct, that this extraordinary man exhumed the recipies which are to cure consumption, and all other diseases of the lungs! How clever it is, thus, to compel the tomb to give up the knowledge which enables the *doctor* and his agents, at the corner of 'Sixth and Chesnut streets,' Philadelphia, to disappoint its future expectations! This is fairly turning the tables upon the grave. But we must no longer trifle with the curiosity of our readers; therefore, let the doctor speak for himself:—

"DESCRIPTION OF THE PULMEL.

"It is a peculiar compound substance, formed by the chemical combination of several powerful vegetable principles, acting on the lungs and the whole system. It contains no pernicious nor poisonous substance. The taste and smell are sweet, fragrant, and balsamic.

1. *Syrup* for internal use.
2. *Balsam* for inhalation, both liquid and solid.
3. *Balsamic Syrup*, that may be used in both ways, internally and for breathing.

4. *Lotion* or *Milk* of *Pulmel*, for external use as a wash, for frictions, and to inhale the fragrant smell.

5. *Wine of Pulmel*, for general use in debility, made with sweet, fragrant, and healthy wines.

6. *Sweet Chocolate of Pulmel*, in cakes, for internal use.

7. *Liquid ditto*, in bottles, merely requiring to be mixed with warm water or milk to make a cup of chocolate instantly.

8. *Sugar of Pulmel*, for internal use; to be used like common sugar, in milk, tea, coffee, or chocolate.

9. *Honey of Pulmel*, to be used like the Sugar, or eaten with bread.

10. *Lozenges of Pulmel*, for the dry cough, sore throat, and painful consumptions.

11. *Powders of Pulmel*, for internal use; may be sent by mail. Dose six grains.

12. *Pulmelin*, or Concentrated Salt of Pulmel, for internal use; easily sent by mail. Dose one grain, but double price."

The *doctor's* circular is embellished with a handsome vignette on wood, having the motto, 'I heal,' addressed no doubt to his patients. This is very well; but, without wishing to be thought officious, we would respectfully recommend, for the next edition, a line, which, if less 'ambrosial' to the people, will be more applicable:—

"Ye *would* be dupes and victims, and ye *are*."

From the important discoveries made by *doctor* Thomson, *doctor* Swaim, *doctor* Rafinesque, and other 'gifted' individuals, it may, perhaps, be thought by some, that the 19th century is peculiarly blessed with great men; but in our desire to do equal justice to every age, we are constrained to say that past times have seen their like. There is now on our table that most learned work, in one volume of 1207 pages, printed in London, 1693, and dedicated to queen Mary II. by William Salmon, Professor of Physic, the title of which is prefixed to this article.

Like *doctor* Thompson, *doctor* Salmon had been 30 years in the practice of physic, before he produced this "short manual of Physick, design'd for the general use of her Majesties subjects, accommodated to mean capacities, in order to the Restauration of their Healths," and like the *reverend* orator Robinson, he inquires "Why should the name of COLLEGE bear sway against your MAJESTIES and the KINGDOMS Interests? Or why should not a private man, who possibly may have abilities for such an undertaking, be heard, because he wears not Titles of Honor and Grandure?" Finally, like the modern author of the "New Guide to Health or Botanical Family Physician," *doctor* Salmon was obliged to strike out a new course. "I have, says he, laboured in an untrodden Path, and brought forth a Work, the very naming of which alone, were almost enough to affright one from the Attempt. But the thing was to be done, it was necessary to be done, and to be done by some body it must; the public necessity required it, and the general good that such a performance might do to Mankind, were motives to the undertaking."

What a noble pair of brothers these two benefactors would have made; but then what would the world have done, had nature in a freak thrown them both into the same century. But her ladyship is not so malevolent. She distributes the higher orders of quacks through all ages instead of concentrating them into one, that the people of no particular generation may be in danger of extinction.

Having favored our readers with a few extracts from the modern works which have been named, we shall edify them with some specimens of the 'Druggists Shop Opened.' If the comparison should not be in favor of *doctor* Salmon's audacity, it will at least show, that he was the prototype of the 'peoples doctors' of 1830.

Notwithstanding so many points of resemblance between *doctor* Thomson and *doctor* Salmon, the latter opens shop with the mineral substances, which the former so strongly condemns.

"Who shall decide when doctors disagree?"

Undoubtedly the people: the *ultima ratio empiricorum.* Let *doctor* Salmon, then, be heard by the people, before they assign the civic crown to his rival.

"ANTIMONIUM. *Antimony.*

"*A Glorious and most effectual Tincture of Antimony may be thus made:* Take our Philosophick Oil (which we have discoursed of in our *Phylaxa Med. Lib. 2. Cap.* 38.) ℥viij. Antimony in fine Powder ℥iij. digest in a Sand heat for a week, shaking the Glass once a day; then decant the clear: So will you have a most glorious Tincture of Antimony not inferiour to any other, nor much inferiour to the Tincture of Gold. It is good against all manner of Fevers, Continent and Continual, whether Putrid, non-Putrid, or Pestelential, being certainly one of the greatest Cordials that can be made by humane Wisdom or Industry. It revives the Spirits, chears the Heart, restores the radical Moisture, and comforts to a miracle Languishing Nature. Dose *a gut. ad* lx. in any proper Vehicle."

"ARGENTUM VIVUM.—*Quicksilver or Mercury.*

In regard to this, our author differs still more widely from *doctor* Thomson; but coincides with *doctor* Swaim, whose *mercurial* Panacea has been lauded, for nearly the same ailments that are set forth in the following extracts:—

"It is said to be cold and moist in the second degree. *Cæsalpinus* saith, that, taken inwardly, it cleanseth the Bloud from filth, it kills and expells Worms. And 'tis certainly true, that it dissolves all manner of Coaggulations and Congealations of the Bloud and Humours, rectifies the Discrasie of the Bloud, purifies even the Marrow in the Bones, and resists all manner of Putrefaction and Malignity in the whole Body proceeding either from Bitings of venemous Creatures, Poison, Plague, &c.

"Outwardly it helps all kinds of Itch, kills Lice, and dissolves hard Tumours in any part of the Body, though in the Joints, eases all manner of pains almost to a Miracle, resolves Nodes, Tophs, and Gums proceeding from King's-Evil, or the like, and has been found of Mighty use against Cancers of all sorts, whether Ulcerated or not. Some Authors say, that being worn about the Neck as an *Amulet* or *Xenecton*, it preserveth from the Plague, as also from Inchantments and Witchcraft. There is no particular Simple in the Shops, except Antimony, that will yield greater Variety of Remedies than Mercury, nor of which more various preparations are made, serving to Purge, Vomit, Sweat, Salivate, ease Pain, cleanse Wounds, heal Ulcers, remove Deformities of the Skin, &c."

"ARGENTUM, *Silver*.

To the following opinions of *doctor* Salmon, on the efficacy of *silver*, we confidently expect a feeling assent by the whole body of the people. After giving his readers the Hebrew, Greek, and Latin names of this celebrated mineral, *doctor* S. proceeds to say:—

"It is cold and moist, and Cephalick, and therefore is said to strengthen the Head; and by chearing the animal Spirits, resists all diseases of that part, chiefly the Megrim, Apoplexy, Epilepsie, Vertigo, and other like diseases akin to them.

The Tincture prevails also against the Palpitation or trembling of the heart, and in some measure has the Virtue of Tincture of Gold, but not altogether so powerfull: It prevails against Madness, Melancholy, and other Distempers of the Brain, comforting and strengthening the same."

---

TO BE CONCLUDED AT PAGE 455.

We owe an apology to such of our readers, as reside beyond the region of epidemic steam quackeries, for occupying so large a part of our present number with this *badinage*. Having begun the printing of the article 'Miscellaneous Intelligence,' before this Review was prepared, and having unconsciously extended it beyond the space allotted to it, we are compelled either to amputate its caudal extremity, or to finish it under our analytical head, and have ventured on the latter alternative. To such as may be displeased with its length, we can only say, that we are with them, in opinion and feeling; and promising not to sin again in the same way, indulge the hope being forgiven. EDITOR.

### THE PEOPLE'S DOCTORS. A REVIEW.

[Concluded from page 420.]

"AURUM, *Gold.*

But the virtues of the *rex metallorum*, although of the same kind, are incomparably greater than those of silver:

"It is Cordial, mundyfying, strengthening the Heart, chears the Spirits, strengthens the natural balsam of Blood, and therefore, is given successfully in all Diseases, wherein the Heat, Life, Vivacity, and Strength of both Body and Spirits need repairing; it is Sudorifick, cleanses the Bloud, discusses Humors, and resists the Poison of Mercury, and other mineral Fumes.

"And as its Tincture, given in Wine to ten or twenty drops, does revive those that are at Deaths-door; so also it is very good against the falling-Sickness, Apoplexy, Palsy, Vertigo, Megrim, Lethargy, and other Diseases of the Head, and destroys the Root and Seminaries of all malignant and poisonous Diseases: It resists Putrefaction and is prevalent against the Plague, and all sorts of pestilential Diseases."

We recommend the king of metals to the afflicted, but cannot speak from experience, as we never yet had enough to make the trial. Even the steam-doctors themselves will scarcely gainsay this advisement; and we think it extremely probable, that however reluctant they may be to *give*, they will not often show many qualms about *taking* the precious metals.

"MARGARITA, *Pearl.*

Many of our readers will recollect, that a few years ago, when Prof. Rafinesque condescended to sojourn in the Backwoods, he proposed to our capitalists, to undertake the growing of *pearls*; but their money was employed at that time in growing *hogs;* and, as they did not think it right to place *pearls* before *swine*, they declined the Professor's proposal; and he departed for Philadelphia, as a better theatre for his scientific enterprises. At that time, the wisest of us did not fully understand what the professor would be at; but it is clear to us now, that being a great antiquary, he had read the following notice, in *doctor* Salmon; and would have cured his patients with *pearls* instead of *Pulmel*, if his learning had been properly appreciated by our monied men.

"Authors say Pearls are cold and dry in the second degree, but I

believe them to be rather temperate in respect of those Qualities; they prevail against Fainting, Swooning, Palpitation of the heart, and all other cardiac Passions, and cure Heart burnings in a moment by absorbing the Acid Humor; they chear the Heart, revive the Spirits, comfort Nature, and restore Strength lost; they stop all sorts of Fluxes, as Diarrheas, Lienteries, and Dysenterias, as also the Hepatick Flux. *Aldrovandus* saith, the levigated Pouder is good to be put into *Collyriums* for the Eyes, for that it takes away the sharpness of the Humor, takes away Clouds, Films, and Dimness, cleanses them from Filth, and strengthens the Nerves by which moisture flows into them. Taken inwardly *ad* ℥j. it removes Melancholy, and heavy pressures of the Spirits, defends against pestilent Diseases. *Mylias* saith, it stops the toothache and cleanseth the Teeth, and is mixed with other Cordial Remedies; It is also prevalent aginst Fevers and all preternatural heats; put upon sharp corroding eating Ulcers, and where the Nerves are bare, it does much good, and gives present ease in the pains thereof. They are by all accounted an excellent Cordial, by which the oppressed Balsam of Life, and decayed strength are re-created and fortified, as *Paracelsus* affirms."

Now here an important question arises, and will perhaps never be answered. It is, whether *doctor* Rafinesque would not have killed fewer people, (oysters excepted) if he had engaged in the practice with pearls, than will be destroyed by his 17 preparations of Pulmel.

But we must hasten into other compartments of the 'Druggist's Shop.'

We have already intimated, that *doctor* Thomson has found RED CLOVER a *cure* for *Cancers*. The following is his formula:—

"CANCER PLASTER.

"Take the heads of red clover and fill a brass kettle, and boil them in water for one hour; then take them out and fill the kettle again with fresh ones and boil them as before in the same liquor. Strain it off and press the heads to get out all the juice; then simmer it over a slow fire till it is about the consistence of tar, when it will be fit for use. Be careful not to let it burn. When used it should be spread on a piece of bladder, split and made soft. It is good to CURE cancers, sore lips, and old sore:"

In justice to *doctor* Salmon, and to compare the great and 'gifted' men of different centuries, we shall transcribe a part of *his* remarks on this rare and powerful medicine:—

"It is temperate, drying, astringent and strengthening; eases fretting pains of the bowels and brings forth sharp and slimy humours; is an excellent vulnerary whether inwardly or outwardly used; and mixt with Honey and dropt into the Eyes, it eases their pain and inflammation, and takes away the Pinn and Web; and being drunk it cures the bitings of Serpents or other venomous Beasts, heals green Wounds and gives ease in the Gout."

It was doubtless when his genius was pampered by 'living in clover,' seasoned with 'cayenne' and 'brandy,' that orator Robinson burst forth into the following ejaculation, which might be compared to Dean Swift's 'Pious Meditations on a broomstick'—

> "If the immense riches of medical virtue, inherent in the plants and flowers of the field, were collected in one volume, it would realize the aspiring hope of the great and good Dr. Rush, the perfect cure of all the maladies of the human race. And the rays of human thought are converging on this sublime, and grand, and awful elevation—the perfection of the healing art; and will continue to concentrate their energies until the full blaze of glorious triumph shall burst upon the world."

We have quoted *doctor* Thomson's opinion of the virtue of *chicken* broth, but he seems to be ignorant of the still greater efficacy of *turkey-buzzard* broth. The following are the words of *doctor* Salmon:

> "*The Flesh* is seldom eaten, but the Broth thereof, and the substance thereof eaten, are said to cure the Leprosie, Elephantiasis, Swellings, Tumors, King's-Evil, Botches, Boils, Scabs, malignant Herpes, Tettars, or Ring-worms, Gout, Epilepsies, Convulsions, Palsies, Morphew, Elephantiasis, Struma, Spitting of Blood, and *helps dimness of sight.*"

In reference to the last important object, we would respectfully advise the people to try this broth for awhile, before they resort to our Eye Infirmaries, where the regular doctors may poison them with 'marcury' and 'bleeding.' If it should fail, they might have recourse to the following recipe, of which *doctor* Salmon seems to have a still higher opinion:

> "*Ashes of a Cat's Head* ʒj. *white vitriol in fine Pouder,* Saccharum Saturni *ana* ℈j. *mix them for a Pouder;* or mix them with Honey for a Balsam, blown into the Eyes, or annointed thrice aday; it cures Blindness, and most Diseases of the Eyes, as the Pin and Web, Pearls, Clouds, Films, &c."

"An Elixer Universall; *Not particular for any Distemper.*

"Rex Metallorum (Gold) ʒss.

Pouder of a Lyons Heart ʒiv.

Filings of a Unicorn's Horn ʒss.

Ashes of the whole Chameleon ʒiss.

Bark of the Witch-Hazle, two handfuls.

Earth Worms (Lumbrici) a score.

Dried Man's Brain ʒv.

Bruisewort (Saponaria) } Each ℔ss.

Egyptian Onions }

"Mix the ingredients together and digest in *my Spirits Universalis*

with a warm digestion, from the change of the Moon to the full, and pass through a fine strainer.

'This Elixer is temperately hot and moist, Digestive, Lenitive, Dissolutive, Aperative, Strengthening, and Glutinative: It opens obstructions, proves Hypnotick and Styptick, is Cardiack and may become Alexpharmick. It is not specially, great, for any one single Distemper; but of much use and benefit in most cases, wherein there is difficulty and embarrassment, or that which might be done, doth not so clearly appear manifest and open to the Eye."

We have copied nothing out of *doctor* Salmon with greater pleasure than the foregoing receipt. It is exactly what is wanted, both by the people and the doctors. As a family medicine it would be unrivalled; for being, to some extent, adapted to all diseases, it might in most cases do a certain amount of good, and Nature could finish the cure. Thus the necessity of employing a physician would often be wholly prevented; a *desideratum*, with the people, which every people's friend should labor to supply. It might, moreover, enable them to correct the injury which is done to their health by nostrums; and, thereby, afford them the pleasure of remaining still longer in the hands of quacks. To dyspepticks and hypochondriacks it would be a treasure; as they might frequently resort to it, when they feel bad and fidgetty, and don't know exactly what to be at. To the *bon vivans* and *gourmands*, it would not be of less value, for a dram of this Elixir would prepare their stomachs for the comfortable reception of the turtle soup, Welch rabbit, egg-nog, champaign, and fourth proof brandy, which 'good company' dooms them to ingurgitate.

To the people's doctors themselves, it could be made equally useful; as, in every disease, it gives *some* relief, which is all that is necessary to establish them in the confidence of the people. Indeed, we think it not unlikely, that some 'natural' will soon take out a patent for this receipt, and convince the people, that it is not merely a palliative, as *doctor* Salmon has modestly set forth, but an infallible cure for all distempers—to which end, judging from the past, it would not be difficult to get a large number of certificates, either attested before a justice of the peace, or certified on honor.

Even to the regular Doctors it would not be without its value, as it would come to their aid on many trying occasions. They should, indeed, never be without it in their pockets, for they know not at what moment a case may offer, which they have forgotten how to treat, or never knew; when it might be made a sort of 'tub to the whale,' for the patient and his friends, until they could go home and read. It would be equally adapted to the cases which they have not time to inquire into, without abridging their hours of idleness and gossip; as, also, to all such as are obscure and undefinable; about which they must never betray any doubts to the people, if they would retain the people's confidence, for the people tolerate no doubters in the practice of medicine: They wisely suppose, that their 'life assurance' is strong, in proportion to the assurance of the doctor; and where the latter is wanting, are apt to think the former defective. Finally, there are several diseases, which generally prove fatal, (except when treated by *doctor* Thompson,) and in these it might be well to try this Elixer of all virtues, as the least troublesome method of investigation and discovery.

Since we are engaged, for the benefit of the people, in extracts from this great repository of one of the people's *doctors*, we shall copy the following, which we would advise our Backwood's friends, who live where foxes are plenty, to try before they send to Philadelphia for *doctor* Rafinesque's *pulmel*, for the cure of consumptions:

"An Extract or Electuary of *Fox Lungs* is reported to be good against Coughs, Colds, Asthma's, all manner of Obstructions of the Lungs, shortness of Breath, difficulty of Breathing, &c. And for these Purposes the Lungs of a *Hart*, *Buck*, or *Doe*, are yet preferable; now if the gross Body of the Lungs will do this, what may be supposed the *Volatile Salt* will do? Truely, it will do that in *three minutes*, which the other will not so well do in 10 *days;* it will do all the aforementioned things, and cure an *exquisite Pleurisie* upon the Spot, this I speak by experience.

"I must intreat the excuse and pardon of the Galenick Physician, and many others of the learned World for my being so zealous for the use of Volatile Animal Salts, since I have had a large practical Experience of their admirable, yea, their stupendious effects, for the best part of thirty years last past; I speak not from the Opinions or Assertions of other Men, I only assert naked Truths from a continued Series of Experiences for near thirty Years together."

The close of this important annunciation, recals to our minds the following sentences in *doctor* Thomson:—

"Long has a general medicine been sought for, and I am confident I have found such as are universally applicable in all cases of disease, and which may be used with safety and success, in the hands of the people.

"After thirty years study and repeated successful trials of the medicinal vegetables of our country, in all the diseases incident to our climate; I can, with well grounded assurance, recommend my system of practice and medicines to the public, as salutary and efficacious."

These two great men seem indeed to have innumerable points of resemblance. *Doctor* Salmon, however, like *doctor* Rafinesque, and unlike *doctor* Thomson, was a man of learning, and gave a much wider scope to his inquiries. Thus in the following article he displays an extent of research, and philosophical acuteness, which *doctor* Swaim, and all the *steam-doctors*, may envy, but can never hope to reach:—

CRANIUM HUMANUM, *Man's Skull.*

"It is called in Hebrew, *Gulgoleth Meta:* in Greek, *Kranion Anthropou*: in Latin, *Cranium humanum*: in English, *Man's Skull.*

Whether it ought to be the *Skull* of a *Man* dying a violent death, or a Natural, is not much material; because the cause of a Natural death, never proceeds from a decay or hurt of the Principles of which the Bones are composed, but rather from a decay of the fleshy Organs and Viscera, and a hurt of the Spirits, Vital, and Animal.

"Some Authors are of Opinion, that it ought to be a *Skull* never buried, and the fresher the *Skull* the better; but this is a great doubt, since my own experience has confirmed, that the simple Pouder, made of a *Skull* many years buried, has done more in the cure of Epilepsies, than all the Elaborate Chymical Preparations, made from those which have been fresh and green.

We have oftentimes used *Skulls* unburied without success, whereas we have often used the other with profit to the Sick. And in a certain Patient, we had given more than 30 Doses of *Skulls* not buried, without any success at all; whereas upon the exhibition of 6 or 7 Doses of that which had been long buried, the same Patient, miraculously recovered, and was perfectly restored, so as the Disease never returned any more; and the same thing we have since several times proved in several other Epilepticks to our great satisfaction.

The reason of this thing is almost obvious; for in a fresh *Skull*, the Particles are not so maturated or ripened, so as to set the Volatile Parts at liberty to operate, as they are in a *Skull* long buried, the which may easily be proved in distillation, for that the Volatile Parts, are nothing near so easie to ascend in the former, as they are in the latter.

Moreover the latter is more imbibed, impregnated, or satiated with the Volatile Parts of the Flesh on the outside, and substance of the Brain within, all which putrefying about the *Skull*, and drying away, the substance of the *Skull* becomes meliorated, not only with its own proper Spirits and Salt; but is also much better digested, by being, as it were, imbibed or immersed in the Humidities and Volatile Particles of the Parts adjacent, which putrefying about it, and leaving their pure Parts at liberty, are probably attracted and drawn into the substance of the Bones of the *Skull.*

I offer this, as something of Reason, joined with Experience, yet not willing to impose upon any Man's credulity, farther than the power of truth, and matter of Fact shall prevail.

From *Man's Skull* several Preprarations are made, as. 1. A Levigated Pouder. 2. A Magistery. 3. The *Galreda Paracelsi.* 4. A Tincture with Spirits of Wine, Juniper or Sage. 5. A Tincture made with our *Spiritus Universalis.* 6. The Spirit, Oil, and Volatile Salt. 7. The fixed Salt. 8. The Essential Powers. 9. The *Protestates Nitratæ*; of all which in order."

These nine preparations of skull seem to have suggested to *doctor* Rafinesque his twelve preparations of pulmel.

Indeed the further we look into the 'Druggist's Shop Opened' by the great people's *doctor* of the 17th century, the more we are convinced, that it is the magazine from which the people's *doctors*, of the present age, have drawn most of their learning. It contains some things, to be sure, which are not quite current at this time, such as witchcraft and spells; but these have still their advocates among us, and at the period when *doctor* Salmon lived, the majority of the people believed in their existence. On the whole, the 'Druggist's Shop Opened' of 1693, approached altogether as closely to the books of the profession at that time, as the 'Botanical Family Physician' of 1828, does the medical writings of the present day.

In asserting the claim, of *doctor* Salmon to the paternity, of much which the people's doctors are now imposing on them as *new*, we perform, as reviewers, but an act of common justice; the more loudly called for, as *doctor* Salmon had no orator Henley, or orator Robinson, to trumpet forth his fame. He seems, however, not to have been altogether incapable of indicting an encomiastic paragraph on his own labours, which we are compelled to say is quite as applicable to them, as are the inflated periods of the *steam orator*, to the labors of the *steam-doctor*. The following is the closing sentence of his book, and will finish our extended notice of it:

"Thus at length with much labour and pains, I have gone through and performed this so exceeding needful, so much desired, so long waited for work, wholly new in it its kind, and contrived in a practical method for the publick good, through the help of him, who works and none can let, who makes a way in the Sea, and a path in the mighty Waters, leading the Blind by the Hand, and guiding them in Paths they have not known.

Our extracts from the 'Compleat English Physician,' will, we think, be sufficient to rescue the reign of William and Mary, from any charge of defective originality; while they serve to show, that great as may be the fooleries of *doctor* Thomson, still greater had been previously invented. They differ, however, *only* in degree. In all ages, quackery has been essentially the same—a compound of ignorance, effrontery, falsehood, and libels upon science and virtue. Every quack is, indeed, a *demagogue*; and relies, for his success, on nearly the same arts, with his political and religious, or rather *irreligious*, brethren. He is one of the *people*, and pre-eminently the guardian of the *people;* while those who spend their lives, in acquiring the knowledge which has been handed down by the great physicians and benefactors of the world, are not *of* the *people*, but arrayed against the *people*, and bent on killing them off with rats bane, as if they were no better than so many Norway rats! Thus it is that the *people* allow themselves to be *charmed*, till they lose their senses, and crawl into the serpent's mouth. Would you arrest them, you thrust yourself between a snake and a fool—to be hissed into wonderment by the former, and brayed into silence by the latter. Happily, the injury you receive, is as little as the good you can do. But if you can do no good, why should you attempt to interpose? Or, why devote to the quack and his victims, so many pages of your Journal? This is a knotty question, and we shall leave our readers to answer it for themselves; content if the bill of fare we have served up, shall have, in any degree, relieved them from the fatigue of weightier studies.

